# From Elixirs to Geroscience: A Historical and Molecular Perspective on Anti-Aging Medicine

**DOI:** 10.3390/molecules30244728

**Published:** 2025-12-10

**Authors:** Giuseppe Rosario Pietro Nicoletti, Katia Mangano, Ferdinando Nicoletti, Eugenio Cavalli

**Affiliations:** Department of Biomedical and Biotechnological Sciences, University of Catania, 95123 Catania, Italy

**Keywords:** anti-aging medicine, geroscience, cellular senescence, nutrient-sensing pathways, mTOR, sirtuins, metformin, senolytics

## Abstract

The pursuit of youth and longevity has accompanied human societies for millennia, evolving from mythological and esoteric traditions toward a scientific understanding of aging. Early concepts such as Greek ambrosia, Taoist elixirs, and medieval “aqua vitae” reflected symbolic or spiritual interpretations. A major conceptual transition occurred between the late nineteenth and early twentieth centuries, when aging began to be framed as a biological process. Pioneering ideas by Metchnikoff, together with early and sometimes controversial attempts such as Voronoff’s grafting experiments, marked the first efforts to rationalize aging scientifically. In the mid-twentieth century, discoveries including the Hayflick limit, telomere biology, oxidative stress, and mitochondrial dysfunction established gerontology as an experimental discipline. Contemporary geroscience integrates these insights into a coherent framework linking cellular pathways to chronic disease risk. Central roles are played by nutrient-sensing networks such as mTOR, AMPK, and sirtuins, together with mitochondrial regulation, proteostasis, and cellular senescence. Interventions, including caloric restriction, fasting-mimicking diets, rapalogues, sirtuin activators, metformin, NAD^+^ boosters, senolytics, and antioxidant combinations such as GlyNAC, show consistent benefits across multiple model organisms, with early human trials reporting improvements in immune function, mitochondrial activity, and biomarkers of aging. Recent advances extend to epigenetic clocks, multi-omic profiling, gender-specific responses, and emerging regenerative and gene-based approaches. Overall, the evolution from historical elixirs to molecular geroscience highlights a shift toward targeting aging itself as a modifiable biological process and outlines a growing translational landscape aimed at extending healthspan and reducing age-related morbidity.

## 1. Introduction

The pursuit of prolonging youth and delaying aging has accompanied human history, blending symbolic traditions with early medical attempts. Ancient cultures imagined aging as a challenge to overcome through mythical elixirs such as Greek ambrosia, Taoist immortality potions, and medieval “aqua vitae”, while Egyptian cosmetics, Galenic dietary regimens, and Renaissance remedies reflected the limited biomedical understanding of their times [1,2].

A major conceptual shift occurred between the late nineteenth and early twentieth centuries, when aging began to be framed as a biological process open to scientific investigation. Elie Metchnikoff’s ideas on “autointoxication” and the role of fermented milk in promoting longevity offered one of the earliest mechanistic hypotheses linking physiology, microbiota, and aging, whereas practices like Voronoff’s gonadal xenotransplantation illustrated the enthusiasm and pitfalls of early rejuvenation research [3,4]. Subsequent advances in cellular biology, including the discovery of replicative senescence, telomeres, and telomerase, provided a more rigorous foundation for understanding aging as a cellular and molecular phenomenon [5].

These developments laid the groundwork for modern gerontology and the emergence of geroscience, which connects fundamental aging mechanisms with age-related disease onset [6]. Central to this framework are nutrient-sensing pathways such as mTOR, AMPK, and sirtuins, which regulate metabolic homeostasis, autophagy, and stress responses. Pre-clinical studies in model organisms have shown that interventions including caloric re-striction, fasting-mimicking diets, mTOR inhibition, and sirtuin activation can extend lifespan and healthspan [7,8].

While human translation is underway through clinical research on rapalogues, metformin, NAD^+^ boosters, senolytic combinations, and antioxidant strategies like GlyNAC [9,10,11].

Alongside molecular interventions, advances in epigenetic clocks, multi-omics bio-markers, and sex-specific physiological responses are shaping a more personalized vision of longevity medicine [12,13]. Ethical questions regarding medicalization, accessibility, and the commercialization of anti-aging interventions, however, remain central to the debate.

This review integrates historical and molecular perspectives to trace the evolution of anti-aging approaches, from mythical elixirs to evidence-based geroscience, and critically examines the translational potential and broader societal implications of strategies aimed at promoting healthy longevity.

## 2. From Esoterism to Science: The History of Anti-Aging

The pursuit of rejuvenation has historically mirrored the cultural, medical, and scientific paradigms of each era. In antiquity, aging was often perceived as a destiny that could be resisted through mystical or ritualistic practices. The soma of Vedic tradition, Greek ambrosia, and Taoist elixirs of immortality exemplify how early civilizations projected their aspirations for eternal youth into mythological narratives [14]. Egyptians emphasized cosmetic and hygienic practices Cleopatra’s famed milk baths are emblematic while classical medicine, from Hippocrates to Galen, linked vitality to balance in diet, lifestyle, and humoral equilibrium [15]. Across non-Western cultures, anti-aging practices evolved along parallel but distinct conceptual lines. Traditional Chinese Medicine linked longevity to the balance of Qi, Jing, and organ systems, with practices such as Qigong, herbal tonics, and alchemical elixirs aimed at nourishing vitality and delaying senescence [16,17]. In Ayurveda, aging (jara) was framed as the progressive loss of ojas, with rejuvenation therapies (rasāyana) employing botanical formulations, dietary regimens, and meditative practices to sustain functional capacity [18,19]. In Japan, Kampo medicine integrated Chinese influences with indigenous Shinto concepts of purification and harmony, emphasizing plant-based preparations to maintain vitality [20]. Indigenous traditions in Africa and the Americas similarly incorporated plant-derived compounds and ritual practices to promote resilience and longevity [21]. Although grounded in symbolic frameworks rather than empirical biology, these traditions collectively highlight a global, cross-cultural pursuit of extended vitality, forming an essential backdrop for the later scientific transition toward biological models of aging.

During the Middle Ages and Renaissance, anti-aging strategies converged with alchemy and early natural philosophy. The search for the aqua vitae or “water of life” symbolized both spiritual purification and biological rejuvenation. Paracelsus, often regarded as the father of toxicology, pursued rejuvenating elixirs, while Avicenna integrated diet, exercise, and hygiene into his medical canon, anticipating principles of preventive medicine [22]. Despite these advances, many remedies, including potable gold, mercury, or exotic herbal concoctions, were ineffective and harmful, highlighting the gap between aspiration and evidence [23].

The nineteenth century marked the transition from speculative remedies to scientific hypotheses. Elie Metchnikoff, Nobel laureate in 1908, advanced the theory of “intestinal autointoxication,” proposing that intestinal microbiota contributed to aging and that fermented milk could prolong life [24]. This shift from mystical to biological reasoning represents a watershed moment in the history of anti-aging medicine. The early twentieth century, however, also saw questionable experiments such as Serge Voronoff’s xenotransplantation of primate testicular tissue into men as a means of rejuvenation, a practice that briefly gained popularity before being discredited [25].

**Figure 1:**
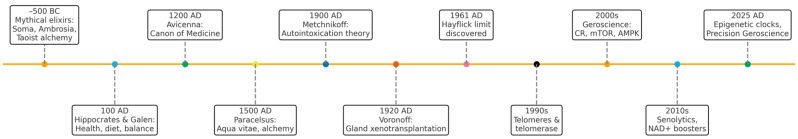
Linear timeline of anti-aging history. Main milestones in anti-aging, from mythical elixirs (soma, ambrosia, Taoist practices) to classical medicine (Hippocrates, Galen), Avicenna and Renasance alchemy (Paracelsus), Metchnikoff’s microbiota theory, and Voronoff’s gland transplants. Key modern discoveries include the Hayflick limit, telomeres, and oxidative stress theory. The 21st century introduced the geroscience paradigm, caloric restriction, mTOR/AMPK/sirtuin pathways, and translational approaches such as metformin, NAD^+^ boosters, senolytics, and GlyNAC. Current focus (2020s–2025) emphasizes epigenetic clocks, omics technologies, and precision geroscience.

By mid-twentieth century, the emergence of cellular biology profoundly transformed aging research. Leonard Hayflick’s discovery of the finite replicative capacity of fibroblasts (the “Hayflick limit”) in 1961 and the identification of telomeres and telomerase in subsequent decades reframed aging as a cellular process [26,27] (Figure 1). In parallel, Denham Harman’s oxidative stress theory (1956) [28] inspired decades of research into antioxidants and vitamins as anti-aging therapies, leading to widespread use despite inconsistent clinical benefits [29].

The twenty-first century inaugurated the era of geroscience, which integrates molecular biology, genetics, and translational medicine. Nutrient-sensing pathways such as mTOR, AMPK, and sirtuins were identified as central regulators of aging, and interventions like caloric restriction, fasting-mimicking diets, and pharmacological modulators (rapalogues, metformin, sirtuin activators) showed lifespan and healthspan extension across multiple model organisms [30,31]. More recent innovations include senolytic therapies targeting senescent cells, NAD^+^ boosters, and antioxidant combinations such as GlyNAC, some of which have entered early clinical trials with promising but heterogeneous outcomes [32,33].

By 2025, the anti-aging field has evolved into a multidimensional discipline. Beyond single interventions, epigenetic clocks, proteomic and metabolomic biomarkers, and gender-specific responses are shaping the next generation of precision geroscience. At the same time, ethical debates around accessibility, equity, and the medicalization of aging remain central, reflecting the persistent tension between the age-old dream of immortality and the modern pursuit of healthy longevity [34].

## 3. Preclinical Evidence: Models of Aging

Research on aging relies on a spectrum of preclinical models that, arranged along the evolutionary axis, provide insights into fundamental mechanisms and allow experimental testing of geroprotective interventions. No single model is universally representative, but comparative use from unicellular organisms to non-human primates has helped to delineate conserved processes and therapeutic targets (Table 1).

Primitive organisms. The yeast *Saccharomyces cerevisiae* remains one of the earliest and most intensively studied systems in aging biology. Its short replicative lifespan and ease of genetic manipulation enabled the discovery of key pathways such as TOR and sirtuins, conserved in mammals and crucial for lifespan and stress resistance [35,36].

Nematodes and insects. The nematode *Caenorhabditis elegans*, with an average lifespan of ~20 days, has facilitated the identification of more than 400 genes that extend longevity, mainly by enhancing resistance to oxidative stress and infections [37]. Its ~60% gene homology with humans and body transparency make it a robust platform for high-throughput screening of senotherapeutics [38,39]. The fruit fly *Drosophila melanogaster* has similarly contributed to the genetics of aging: mutations in the *Indy* gene double lifespan by modulating energy metabolism. More recently, senescent glial cells have been described in the aging fly brain, contributing to cognitive decline and offering insights into neural aging [40,41].

Fish and non-mammalian vertebrates. The African turquoise killifish (*Nothobranchius furzeri*), one of the shortest-lived vertebrates, reaches sexual maturity within weeks and lives only 4–6 months, making it a valuable platform for rapid testing of anti-aging drugs [42,43]. Zebrafish (*Danio rerio*) and axolotls (*Ambystoma mexicanum*) are increasingly used to study how regeneration and senescence intersect in vertebrates. In zebrafish, transient senescent cells appear during tissue repair, such as fin or heart regeneration, where they help shape the local environment and promote recovery [44,45]. Axolotls, with their extraordinary ability to regenerate entire limbs and organs, show that senescence can actively support blastema formation, providing proliferative cues for tissue replacement [46]. These findings highlight that senescence is not only linked to aging but is also an evolutionarily conserved program contributing to development and repair, making these species valuable models for translational insights into regenerative medicine [47].

Rodents. Mice and rats are the cornerstone of geroscience. In these models, caloric restriction robustly extends both lifespan and healthspan [48]. Pharmacological inhibition of mTOR with rapamycin, as well as genetic modifications of GH/IGF-1 signaling, have further demonstrated conserved mechanisms of longevity [49,50]. Rodent models also provided the first causal evidence that senescence drives aging: clearance of senescent cells in transgenic p16^Ink4a^ or p21^Cip1^ mice improved frailty, preserved organ function, and delayed multimorbidity [51,52]. Recent approaches have emphasized using aged animals, both sexes, and genetically diverse strains such as UM-HET3 and Collaborative Cross mice, to improve translatability [53,54,55]. Moreover, innovative paradigms such as polypharmacy models, multimorbidity frameworks, and chronic social stress paradigms are being used to replicate the complexity of human aging [53,56,57].

Swine. Swine, and particularly mini-pigs, are emerging as valuable models in the field of aging and geroscience, owing to their strong physiological and anatomical similarity to humans [58]. Unlike rodents, pigs display metabolic, cardiovascular, and immune system features that more closely parallel human aging trajectories, making them especially relevant for translational studies in longevity research. Mini-pigs have been used to explore age-associated disorders such as atherosclerosis, obesity, and type 2 diabetes, conditions that mirror the metabolic and vascular decline observed in elderly humans [59,60]. Their intermediate lifespan, combined with their susceptibility to age-related pathologies, allows researchers to investigate both the onset and progression of senescence in a clinically meaningful framework. Furthermore, because of their heart size, vascular architecture, and hemodynamics, pigs provide an excellent platform for testing interventions aimed at delaying cardiovascular aging, including pharmacological agents, dietary strategies, and novel medical devices [61,62]. The feasibility of applying human-like surgical, imaging, and monitoring procedures strengthens their role as a bridge between basic research and clinical geroscience. By enabling the study of systemic aging processes in a physiologically relevant organism, swine models are helping to close the gap between experimental anti-aging strategies and their translation into human therapies [63,64].

Non-human primates. Non-human primates are evolutionarily closest to humans. Decades-long caloric restriction studies in rhesus macaques showed improvements in metabolic health, immune function, and delayed onset of diseases, though results on lifespan remain mixed [65,66]. The common marmoset (*Callithrix jacchus*), with its shorter lifespan of 5–7 years, is now used in aging studies, showing pathologies that mirror human age-related conditions [67,68,69].

The progression from yeast to primates illustrates how fundamental mechanisms of aging including metabolic regulation, stress resistance, and cellular senescence are conserved across evolution [70]. Yet, maximizing translational impact requires models that incorporate genetic diversity, environmental complexity, and multimorbidity. Collectively, these models provide a compelling case that aging is malleable and amenable to interventions aimed at extending healthspan.

In addition to classical animal and cellular models, human stem-cell-derived organoids have recently emerged as powerful platforms to model aging processes, including stem-cell exhaustion, DNA damage and senescence-associated changes, and present increased translational value over traditional animal models [71,72].

Importantly, stem cell exhaustion is now recognized as a core hallmark of aging, contributing to impaired tissue homeostasis, reduced regenerative capacity, and increased vulnerability to age-related diseases. Human stem cell–derived organoids provide a powerful platform to model age-associated stem cell dysfunction, senescence, and regenerative decline in a translationally relevant context.

## 4. Molecular Pathways of Aging and Intervention

The molecular breakdown of aging has unveiled highly conserved regulatory networks, particularly mTOR, AMPK, and sirtuins (SIRT1–7) that function as pivotal “master regulators” of lifespan and healthspan [73]. These nutrient-sensing pathways interpret environmental cues, metabolic states, and stress signals to maintain cellular homeostasis, growth, and survival. Understanding these mechanisms reframed aging as a modifiable biological process and paved the way for pharmacological interventions targeting age-related decline [74].

Recent studies have highlighted that nutrient-sensing pathways such as AMPK, mTOR and sirtuins intersect with the epigenetic machinery and influence the trajectory of epigenetic aging. AMPK activation promotes chromatin remodeling and stabilizes DNA-methylation patterns through metabolic sensing mechanisms, while sirtuins modulate histone acetylation and NAD^+^-dependent deacetylation processes that directly impact the epigenetic clock. Furthermore, caloric restriction–related suppression of mTOR signaling has been associated with favorable chromatin accessibility changes and shifts in DNA-methylation age. Together, these findings support a mechanistic interface between metabolic pathways and epigenetic biomarkers of biological aging [75,76].

Among these regulators, the mechanistic target of rapamycin (mTOR) plays a central role. It is a serine/threonine kinase that forms two distinct complexes, mTORC1 and mTORC2, which govern growth, protein synthesis, autophagy, metabolism, and cytoskeletal dynamics [77]. By sensing amino acids, energy levels, and growth factors, mTOR orchestrates cellular anabolic responses [74]. Pharmacological inhibition of mTOR with rapamycin has repeatedly demonstrated lifespan extension across species. A comprehensive review from 167 papers covering eight different vertebrate species shows that rapamycin confers longevity benefits comparable to severe calorie restriction [78], and the Interventions Testing Program (NIH) highlighted rapamycin as the most potent longevity intervention among 13 candidates [79]. Recent studies further confirmed that long-term intermittent rapamycin administration in mice improves metabolic health and promotes intestinal autophagy, yielding sustained geroprotective effects [80]. Translational potential is also suggested by human data: in a 48-week study, low-dose intermittent rapamycin regimens in otherwise healthy aging adults improved lean muscle mass and well-being with acceptable safety [81]. Still, systematic reviews caution that enthusiasm may be outpacing robust evidence, particularly with regard to off-label use in healthy individuals [82].

The search for synergistic effects has also driven interest in combinatorial approaches. A groundbreaking mouse study demonstrated that rapamycin combined with trametinib, a MEK inhibitor, produced additive lifespan extensions of up to ~30% while delaying tumor growth and reducing systemic inflammation [83]. Average longevity increased by around 29% in females and 27% in males, substantially more than with either drug alone [84].

If mTOR represents a sensor of growth and nutrient abundance, the AMP-activated protein kinase (AMPK) operates as the central cellular energy sensor, activated under low-energy states such as fasting, caloric restriction, or exercise. Upon activation, AMPK promotes catabolic pathways to generate ATP, inhibits anabolic processes including mTORC1 signaling, enhances autophagy, and supports mitochondrial function via PGC-1α [85]. Importantly, AMPK interacts with the sirtuin network through NAD^+^-dependent regulatory loops, highlighting an integrated axis of energy and epigenetic regulation [86].

Sirtuins, particularly SIRT1, SIRT3, and SIRT6, are NAD^+^-dependent deacetylases and ADP-ribosyltransferases that govern epigenetic status, stress resistance, metabolism, and DNA repair. SIRT6, for example, is essential for genomic integrity by facilitating DNA double-strand break repair, and its absence induces premature aging phenotypes [87]. Recent reviews have highlighted both natural and synthetic sirtuin activators, such as resveratrol, as promising anti-aging agents, emphasizing their mechanistic properties and geroprotective outcomes [88]. Other analyses underscore their impact on cardio-renal aging and interconnected organ dysfunction [89]. Since declining NAD^+^ levels with age represent a bottleneck for sirtuin activity, supplementation with NAD^+^ precursors such as nicotinamide riboside (NR) and nicotinamide mononucleotide (NMN) has attracted significant attention, rejuvenating mitochondrial function in preclinical models and showing early efficacy in human studies [90].

**Figure 2:**
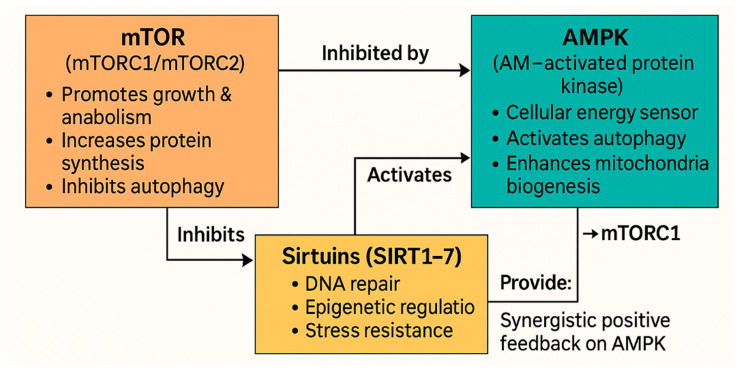
Interactions between the major nutrient-sensing pathways involved in aging. AMPK inhibits mTORC1 signaling and promotes autophagy and mitochondrial biogenesis (→). AMPK activation also stimulates sirtuins through enhanced NAD^+^ availability. Sirtuins, in turn, exert positive feedback on AMPK and partially suppress mTORC1 activity through deacetylation-dependent regulation of metabolic and stress-response pathways. mTORC1 promotes anabolic processes, increases protein synthesis, and inhibits autophagy. Arrows indicate activating interactions, while blunt lines represent inhibitory pathways.

These pathways do not operate in isolation but rather form a tightly interconnected regulatory network (Figure 2). AMPK inhibits mTORC1 while enhancing sirtuin activity, and NAD^+^ levels modulate both AMPK and sirtuins, creating feedback loops that align energy and stress responses. This integration helps explain why caloric restriction or fasting engages multiple pathways synergistically [91]. Beyond rapamycin and trametinib, several phytochemicals such as resveratrol, curcumin, and quercetin also modulate mTOR, AMPK, sirtuins, and NF-κB pathways, representing accessible geroprotective strategies [16]. Meanwhile, innovations are underway to create next-generation mTORC1-specific inhibitors, such as DL001 and HY-124798, which aim to minimize side effects linked to mTORC2 inhibition [92,93].

Despite these advances, clinical translation remains in its infancy. While rapamycin is the frontrunner, human applications are still limited and largely preliminary [94]. Proposed indications include delaying menopause or mitigating periodontal disease, but these remain exploratory [95,96,97]. At the same time, novel approaches such as gene therapies that reprogram cells to youthful states or boost NAD^+^-related resilience are increasingly being discussed in the context of geroscience [98]. Together, these discoveries have transformed the perception of aging from an inevitable decline to a biological process that may be actively modulated, opening the door to interventions aimed at extending not only lifespan but also healthspan.

## 5. Cytokines, Inflammaging and MIF

The aging process is increasingly understood as being influenced not only by intrinsic cellular mechanisms but also by a state of chronic, low-grade inflammation, a phenomenon now widely referred to as “inflammaging”. This concept describes the persistent production of pro-inflammatory cytokines in the absence of overt infection, a condition that contributes to tissue dysfunction, frailty, and the development of age-related diseases such as atherosclerosis, diabetes, and neurodegeneration [99]. Among the mediators most consistently implicated in this process are interleukin-6 (IL-6), tumor necrosis factor-α (TNF-α), interleukin-1β (IL-1β), and macrophage migration inhibitory factor (MIF), which together shape the inflammatory landscape of aging. Inflammaging arises through a variety of converging mechanisms, including the accumulation of senescent cells displaying a senescence-associated secretory phenotype (SASP), the activation of innate immune receptors by damage-associated molecular patterns (DAMPs), mitochondrial dysfunction, and dysregulated autophagy [100]. These signals combine to sustain a pro-inflammatory microenvironment that drives systemic aging and accelerates the onset of age-related pathologies.

Within this inflammatory setting, IL-6 assumes a particularly paradoxical role. While acutely beneficial in coordinating immune defense, chronic elevation of IL-6 is strongly associated with sarcopenia, cardiovascular disease, cognitive decline, and increased mortality in the elderly [13]. Persistent IL-6 signaling perpetuates STAT3 activation and contributes to metabolic alterations, most notably insulin resistance. TNF-α, another pleiotropic cytokine produced largely by macrophages and adipocytes, further amplifies the aging phenotype by promoting lipolysis, insulin resistance, and endothelial dysfunction. Its chronic presence has been directly linked to muscle wasting and neuroinflammation, positioning it as a major determinant of frailty and age-associated morbidity [101]. Similarly, IL-1β, released in the context of inflammasome activation, amplifies tissue inflammation and plays a crucial role in both metabolic disturbances and neurodegenerative processes. Elevated IL-1β levels contribute to mitochondrial injury and propagate SASP in surrounding cells, creating a vicious feed-forward loop that reinforces inflammaging [102]. Together, these cytokines form the so-called “cytokine signature” of aging, with systemic levels of IL-6, TNF-α, and IL-1β serving as reliable predictors of functional decline and reduced healthspan [103,104]. Emerging inflammatory mediators also contribute to age-associated dysfunction and deserve mention within the inflammaging framework. Among these, interleukin-17 (IL-17) has gained substantial attention as a driver of chronic inflammation in aging [105]. IL-17–producing Th17 cells expand with age and promote tissue deterioration through neutrophil recruitment, disruption of epithelial barriers, and amplification of IL-6 and TNF-α signaling [106]. Elevated IL-17 levels have been linked to sarcopenia, osteoporosis, cognitive decline, and cardiometabolic disorders in older adults [13,107]. Chemokines likewise represent a key but often underappreciated component of inflammaging. CCL2 (MCP-1), CXCL8 (IL-8), and CXCL10 (IP-10) increase progressively with age, mediating monocyte recruitment, vascular inflammation, and immune dysregulation. High circulating CCL2 and CXCL10 levels correlate with frailty, reduced mobility, and increased mortality in the elderly. Collectively, IL-17 and age-associated chemokine signatures integrate with the canonical IL-6/TNF-α/IL-1β axis, shaping a broader and more dynamic inflammatory network that accelerates multiple age-related diseases [108]. Among the diverse inflammatory mediators that drive this process, MIF occupies a unique and particularly intriguing position. Originally described as a T-cell–derived factor that inhibited macrophage migration, MIF has since been recognized as a pleiotropic regulator that influences immunity, metabolism, and tissue homeostasis [109]. Its biological activity is mediated through binding to CD74 and CXCR2/4 receptors, thereby activating ERK and PI3K–AKT signaling pathways and amplifying NF-κB activity [110]. Unlike classical cytokines, MIF is constitutively expressed and can be rapidly released in response to stress, hypoxia, or glucocorticoids, where it functions as a counter-regulator of anti-inflammatory pathways [111]. Evidence increasingly points to a pathogenic role for MIF in metabolic aging: it has been implicated in promoting adipose tissue inflammation, impairing pancreatic β-cell function, and inducing insulin resistance [112]. In the central nervous system, MIF exacerbates amyloid pathology and contributes to synaptic loss, thereby linking chronic immune activation with cognitive decline [113,114]. Furthermore, genetic polymorphisms within the MIF promoter have been shown to correlate with increased susceptibility to cardiovascular disease and type 2 diabetes, underscoring its importance as a determinant of healthy versus pathological aging [112,115,116]. For these reasons, MIF has emerged as a crucial hub at the intersection of immune regulation and metabolic control, and a pivotal mediator of inflammaging and its complications [117,118,119]. However, the causal role of MIF as a biomarker of aging remains to be fully verified, and further longitudinal human studies are required to establish its predictive validity.

**Figure 3:**
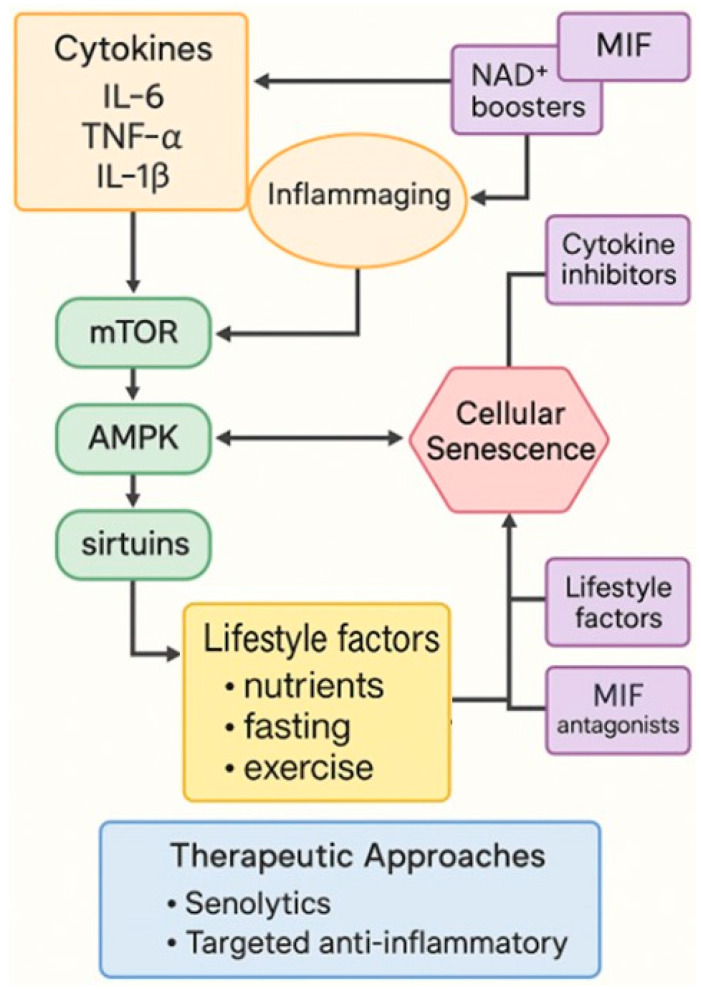
Schematic representation of cytokine-driven inflammaging and its links to mTOR, AMPK, sirtuins, and cellular senescence. Cytokines and MIF promote inflammaging, activating mTOR and contributing to senescence, while AMPK inhibits mTOR and supports sirtuin activity. Senescence feeds back into inflammaging and influences AMPK. Key interventions, including senolytics, cytokine inhibitors, MIF antagonists, NAD^+^ boosters, and lifestyle factors are shown at their primary points of action.

The contribution of cytokines to the biology of aging cannot, however, be considered in isolation from canonical nutrient-sensing pathways such as mTOR, AMPK, and sirtuins. Indeed, these pathways converge with cytokine signaling to orchestrate both cellular and organismal aging [120]. Pro-inflammatory cytokines including IL-6 and TNF-α enhance mTOR activation, thereby driving anabolic responses while suppressing autophagy and promoting senescence [121]. Conversely, AMPK exerts an anti-inflammatory effect by inhibiting NF-κB signaling and supporting mitochondrial biogenesis, although chronic exposure to cytokines impairs AMPK activity and leads to metabolic inflexibility [122]. Sirtuins, and particularly SIRT1, are similarly protective, suppressing cytokine production by deacetylating NF-κB and p53, yet their activity declines with age as NAD^+^ levels fall, which in turn amplifies pro-inflammatory signaling [123]. Interestingly, MIF itself has been shown to interact directly with metabolic checkpoints, modulating glucose utilization, redox balance, and macrophage polarization, thus reinforcing the connection between immune mediators and nutrient-sensing mechanisms [99]. This bidirectional relationship illustrates the systemic nature of aging, where inflammation and metabolism are inseparably intertwined in a network of reinforcing feedback loops (Figure 3).

The recognition of cytokines as central drivers of inflammaging has spurred intense investigation into therapeutic strategies aimed at modulating their activity. One of the most promising approaches is the use of senolytics or SASP modulators, which promote the clearance of senescent cells and thereby reduce the secretion of IL-6, TNF-α, and IL-1β, leading to a measurable reduction in systemic inflammation [32,124]. In parallel, cytokine inhibitors such as monoclonal antibodies and receptor antagonists directed against IL-6 (for example tocilizumab) or IL-1β (for example canakinumab) are being repurposed for use in age-related inflammatory conditions [125,126]. MIF itself has recently emerged as a therapeutic target, with small-molecule inhibitors or CD74 blockade being explored as novel strategies to counter inflammaging and metabolic dysfunction [127,128]. Beyond pharmacological interventions, lifestyle measures such as caloric restriction, regular physical activity, and NAD^+^ supplementation have been shown to indirectly attenuate cytokine production by restoring metabolic resilience, thereby exerting broad anti-aging benefits [129].

Taken together, these observations highlight the central role of cytokine networks as an interface between immune aging, metabolic dysregulation, and tissue senescence. IL-6, TNF-α, and IL-1β define the canonical inflammatory triad of aging, while MIF emerges as a unique regulator that integrates immune and metabolic signals. Their interaction with nutrient-sensing pathways such as mTOR, AMPK, and sirtuins further reinforces the idea that aging is not a linear or unidimensional process but rather a systemic network of interdependent mechanisms that collectively shape the trajectory of healthspan and disease in later life.

## 6. Clinical Trials and Translational Approaches in Geroscience

**Figure 4:**
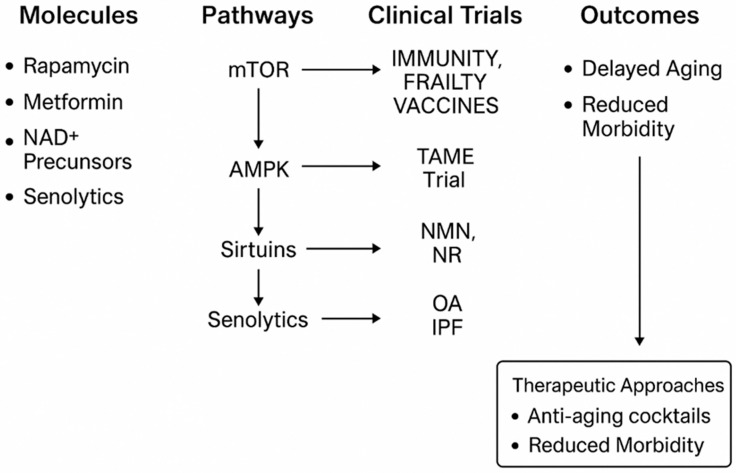
Schematic representation of translational geroscience pathways and clinical trials. Key pharmacological agents (rapamycin, metformin, NAD^+^ precursors, senolytics) act on nutrient-sensing and senescence-related pathways (mTOR, AMPK, sirtuins). These interventions are being tested in human clinical trials, including the TAME study with metformin, everolimus/rapamycin studies on immunity and frailty, NAD^+^ precursor supplementation (NMN, NR), and senolytic pilot trials in osteoarthritis (OA) and idiopathic pulmonary fibrosis (IPF). Expected outcomes include delayed aging, reduced morbidity, and the development of anti-aging therapeutic strategies such as combination “cocktails”.

The transition from preclinical discoveries to human interventions represents one of the greatest challenges in geroscience. While animal models have consistently demonstrated the robust efficacy of caloric restriction, rapamycin, metformin, NAD^+^ boosters, and senolytics in extending both lifespan and healthspan, translating these findings into humans requires careful consideration of safety, dosing, and appropriate clinical endpoints (Figure 4). Unlike traditional clinical trials that focus on single-disease outcomes, geroscience trials aim to evaluate interventions that target the fundamental biology of aging itself, thereby delaying the onset or progression of multiple chronic conditions simultaneously. This paradigm shift raises important methodological, regulatory, and ethical questions regarding how to define success in the context of longevity medicine [6,31].

Among the agents investigated, rapamycin remains the most extensively studied geroprotective compound in animal models, where it has been shown to consistently extend lifespan even when administered late in life [50]. Its translation into humans began with small-scale studies focused primarily on immune function. A pivotal trial demonstrated that low-dose everolimus, a rapamycin derivative, enhanced the immune response to influenza vaccination in elderly adults, suggesting improved immunocompetence [130]. Subsequent investigations have explored intermittent dosing regimens designed to minimize side effects such as stomatitis, hyperlipidemia, or insulin resistance. A 2024 randomized trial reported that once-weekly rapamycin in older adults improved physical performance and lean muscle mass without major adverse events [81]. Importantly, these studies emphasized that chronic daily dosing is not tolerated, highlighting the importance of optimizing schedules that preserve efficacy while minimizing toxicity [82]. Ongoing research is currently assessing the potential of rapamycin for delaying conditions such as periodontal disease, frailty, and even ovarian aging [131]. However, despite these encouraging signals, experts continue to caution against premature enthusiasm for off-label use in healthy populations, given the limited availability of long-term safety data [94].

Metformin, one of the safest and most widely prescribed drugs for type 2 diabetes, has similarly attracted enormous attention for its potential geroprotective effects. Observational studies consistently report a reduced incidence of cancer, cardiovascular disease, and all-cause mortality among metformin users compared to non-diabetic controls [132]. The Targeting Aging with Metformin (TAME) trial, spearheaded by Barzilai and colleagues, represents a landmark effort to reposition an established drug for aging. Unlike conventional trials focusing on a single disease, TAME employs a composite endpoint of age-related morbidities, including cardiovascular disease, cancer, dementia, and mortality [9,133]. Although delayed due to funding and regulatory hurdles, this trial is expected to establish a precedent for how geroscience interventions can be evaluated in humans. Mechanistically, metformin activates AMPK, reduces oxidative stress, improves mitochondrial function, and modulates the gut microbiome [134]. Its excellent safety profile makes it an attractive candidate for long-term preventive use, though questions remain regarding its efficacy in non-diabetic individuals [135].

Another promising class of interventions includes NAD^+^ precursors such as nicotinamide riboside (NR) and nicotinamide mononucleotide (NMN). Declining NAD^+^ levels with age impair mitochondrial function, DNA repair, and sirtuin activity, while supplementation with these compounds has been shown in animal models to restore NAD^+^ pools and improve healthspan [136]. Early human trials have reported mixed but encouraging results: one randomized controlled trial demonstrated that NR supplementation improved skeletal muscle NAD^+^ metabolism and reduced inflammatory markers in older adults [137], while another found that NMN supplementation enhanced insulin sensitivity and muscle remodeling in postmenopausal women with prediabetes [90]. Despite these findings, the clinical effects of NAD^+^ boosters remain modest compared to preclinical results, and larger, longer trials are needed to determine whether these interventions provide durable benefits in reducing morbidity or extending healthspan [138]. In addition, regulatory uncertainty complicates translation, as the FDA recently restricted NMN from being marketed as a dietary supplement in the United States, raising questions about accessibility [139].

The identification of cellular senescence as a key driver of aging has further spurred the development of senolytics, drugs that selectively eliminate senescent cells. Preclinical studies demonstrate that clearance of senescent cells alleviates frailty, preserves organ function, and delays the onset of multiple age-related diseases [140]. The first human pilot study testing a combination of dasatinib and quercetin in patients with idiopathic pulmonary fibrosis showed improved physical function and reductions in senescence-associated biomarkers [11]. Additional trials are now underway in osteoarthritis, chronic kidney disease, and Alzheimer’s disease [141]. Nevertheless, several challenges remain, including the risk of off-target effects, the heterogeneity of senescent cell populations, and the need for reliable biomarkers to monitor therapeutic efficacy. Despite these limitations, senolytics are widely regarded as one of the most exciting frontiers in translational geroscience [142].

Given the interconnectedness of aging pathways, single-drug interventions may ultimately prove insufficient. This has led to growing interest in combination strategies, often referred to as “anti-aging cocktails.” Recent animal studies provide proof-of-concept for such approaches. For instance, a 2025 study from the Max Planck Institute demonstrated that rapamycin combined with trametinib extended mouse lifespan by approximately 30%, a far greater benefit than either agent alone, while simultaneously reducing systemic inflammation and tumorigenesis [83]. Other combinations, such as metformin with NAD^+^ precursors or rapamycin with senolytics, are likewise being explored in preclinical settings. The concept of multi-target interventions mirrors strategies that have been successful in oncology and HIV treatment, reflecting the inherent complexity of aging biology [143]. Translating these regimens into human populations will require a careful balance of efficacy, toxicity, and regulatory acceptance, but they represent a logical next step in the pursuit of effective geroscience-based therapies.

Despite these advances, significant challenges continue to hinder the clinical translation of geroscience interventions. One of the most pressing issues concerns trial endpoints. Unlike disease-specific drugs, which can be evaluated against clear clinical outcomes, anti-aging therapies must demonstrate broad benefits across multiple domains of health. Composite endpoints, such as those adopted in the TAME trial, represent a potential solution, but remain largely unvalidated and continue to spark debate within the field [144]. Another challenge lies in the inherent heterogeneity of the aging population. Older adults present with wide variability in comorbidities, polypharmacy, functional reserve, and lifestyle factors, while additional differences in sex, ethnicity, and genetic background further complicate the design and interpretation of clinical trials [145]. Safety considerations are equally critical, as these interventions are often intended for long-term use in otherwise healthy individuals; under such conditions, even rare adverse events can translate into significant risks. Defining optimal dosing regimens that balance efficacy with safety therefore remains an urgent priority [146]. Regulatory and ethical issues also complicate translation. Because aging itself is not formally recognized as a disease by most regulatory agencies, approval pathways remain uncertain, while broader ethical questions regarding accessibility, equity, and the potential misuse of longevity drugs add further complexity [34].

Looking forward, the future of clinical geroscience is likely to move toward increasingly personalized approaches. The integration of genetic, epigenetic, metabolomic, and microbiome data may allow for the tailoring of interventions to individual biological profiles, maximizing efficacy while minimizing risk. Biomarkers such as epigenetic clocks, proteomic signatures, and immune profiling will be essential both for identifying responders and for tracking therapeutic impact over time [28,147]. In parallel, regenerative strategies are beginning to emerge as complementary avenues to metabolic and immunological interventions. These include stem cell therapies, gene editing technologies, and even partial cellular reprogramming, which collectively point toward a future where longevity medicine becomes a central component of preventive healthcare. Such innovations promise a paradigm shift away from treating established diseases toward preserving resilience across the entire lifespan [148]. Among the clinical initiatives discussed in this section, several studies represent particularly high-impact developments in translational geroscience. These include the TAME trial on metformin, rapalog-based interventions, senolytic trials such as dasatinib plus quercetin, NAD^+^-precursor studies (NR and NMN), and the randomized GlyNAC trial. Together, these representative clinical papers highlight the major current trends in human anti-aging research and reinforce the translational relevance of targeting fundamental aging pathways.

### Pharmacokinetic, Safety, and Translational Considerations of Major Geroprotective Compounds

A detailed assessment of the pharmacokinetics (ADME), clinical dosing, and safety of geroprotective agents is essential to contextualize their translational potential. Although many molecules, rapamycin, metformin, polyphenols, and NAD^+^ boosters, have accumulated substantial preclinical support, human pharmacology often represents the major limiting step for clinical implementation (Table 2).

Rapamycin and rapalogues show relatively low oral bioavailability (10–20%) and a long elimination half-life of approximately 60 h, facilitating intermittent or weekly dosing schemes [149,150,151]. Human pharmacokinetic studies demonstrate extensive hepatic metabolism via CYP3A4 and P-glycoprotein, producing significant interindividual variability [149,152,153]. Clinically meaningful toxicities—including hyperlipidemia, stomatitis, peripheral edema, impaired wound healing, and increased infection risk—remain concerns for long-term use [131,151]. Consequently, emerging longevity-oriented regimens increasingly favor low-dose or pulsatile administration to balance efficacy with tolerability [9,154].

Metformin demonstrates far more predictable pharmacokinetics. It is absorbed through OCT1 transporters, not metabolized, and excreted unchanged renally, with a half-life of 4–9 h [9,133,155]. Standard antidiabetic dosing (1–2 g/day) is generally well tolerated, although gastrointestinal side effects are common, and rare cases of lactic acidosis can occur in renal impairment or multimorbidity [155]. Several studies report metformin’s ability to modulate inflammatory and epigenetic aging markers, strengthening its candidacy for trials such as TAME [133,156].

Polyphenols, including resveratrol, curcumin, and quercetin—have robust preclinical effects but consistently suffer from poor oral bioavailability. Resveratrol undergoes rapid phase II metabolism (glucuronidation and sulfation), leading to plasma concentrations orders of magnitude below those required in vitro [157,158,159]. Curcumin is even more limited, with <1% systemic bioavailability unless administered in lipid nanoparticles or piperine-enhanced formulations [160]. Nonetheless, toxicity remains low, although hepatotoxicity has been occasionally described with high-dose supplements or multi-compound extracts [159].

NAD^+^-boosting compounds (NR and NMN) display promising short-term safety profiles. NR increases blood NAD^+^ within hours and exhibits dose-dependent pharmacokinetics, whereas NMN may depend on transporter SLC12A8 for cellular uptake [153,161]. Human studies up to 12 months report good tolerability; however, concerns remain regarding theoretical cancer risks, metabolic reprogramming, and long-term effects in elderly or multimorbid populations.

Overall, the existing evidence highlights substantial gaps: limited long-term toxicity data, absence of standardized dosing frameworks, and major variability in human bioavailability, particularly for natural compounds. Integration of pharmacokinetic monitoring with biomarkers of target engagement (e.g., mTOR phosphorylation, AMPK activation, NAD^+^ levels) will be essential to advancing these interventions toward safe and effective clinical geroscience.

**Table 2:** Pharmacokinetic, Clinical, and Safety Profiles of Major Geroprotective Compounds.

Molecule	ADME Profile	Typical Human Doses	Adverse Effects	Key References
Rapamycin/Rapalogues	Low oral bioavailability (10–20%); CYP3A4 metabolism; long t½ (~60 h); P-gp substrate	Intermittent 2–6 mg/week; low-dose regimens in aging trials	Hyperlipidemia, stomatitis, wound-healing delay, edema, infection risk	[131,149,151,152,154]
Metformin	Absorbed via OCT1; not metabolized; renal excretion; t½ 4–9 h	1–2 g/day (standard); lower doses in aging trials	GI upset, B12 deficiency; rare lactic acidosis (renal impairment)	[9,133,155]
Resveratrol	Rapid glucuronidation & sulfation; low systemic bioavailability	150–1000 mg/day; enhanced forms up to 2 g/day	Occasional hepatotoxicity at high doses; GI discomfort	[157,158,159]
Curcumin	<1% bioavailability; rapid metabolism; improved with piperine/nanocarriers	500–2000 mg/day; enhanced to 4–8 g/day	GI issues; rare hepatotoxicity (high-dose extracts)	[160]
Quercetin	Poor absorption; extensive metabolism; short t½	500–1000 mg/day in small clinical studies	Headache, GI discomfort; CYP3A4 interactions	[162]
NAD^+^ Boosters (NR/NMN)	NR: good oral absorption, raises NAD^+^ rapidly; NMN: transporter-mediated uptake	NR 250–1000 mg/day; NMN 300–600 mg/day	Generally well tolerated; theoretical cancer/metabolic risks	[153]

## 7. Registered Clinical Trials in Geroscience

Information regarding the status and registration details of clinical trials was retrieved from ClinicalTrials.gov and related registries on 10 January 2025.

The landscape of geroscience clinical trials is expanding rapidly, with several classes of interventions progressing from preclinical validation to human testing. Rapalogues represent one of the most advanced categories, and the EVERLAST trial (NCT05835999) is currently a phase II study investigating the safety and anti-aging effects of everolimus, an mTORC1 inhibitor, administered over 24 weeks in older adults. Building upon earlier evidence that rapalogues can enhance immune function and reduce markers of immunosenescence [130], this trial aims to assess functional outcomes, immune responses, and potential adverse effects in a preventive context. Its primary endpoints include safety, tolerability, and biomarkers of aging-related processes such as autophagy and immune competence [163]. The results are expected to clarify whether intermittent dosing regimens of rapalogues can balance efficacy with minimized side effects in human populations.

Metformin has also been at the center of significant attention. The MILES trial (Metformin in Longevity Study, NCT02432287) was designed to evaluate whether metformin, a widely prescribed antidiabetic drug, modulates biomarkers of aging in non-diabetic older adults. This trial focused particularly on transcriptional changes across tissues such as skeletal muscle and adipose tissue, reflecting pathways linked to inflammation, mitochondrial function, and insulin sensitivity [9]. Although completed, its published outputs remain preliminary, emphasizing biomarker shifts rather than definitive clinical outcomes [156]. Running in parallel, the Targeting Aging with Metformin (TAME) trial, though not yet consolidated under a single NCT identifier, represents a landmark initiative to evaluate metformin across multiple age-related morbidities. Its composite endpoint includes cardiovascular events, cancer, cognitive decline, and mortality [9]. Although delayed by funding and regulatory barriers, TAME has set an important precedent for how anti-aging interventions may eventually be evaluated within formal clinical frameworks.

Another line of research is focused on restoring NAD^+^ metabolism, which declines with age and contributes to impaired mitochondrial function, reduced DNA repair, and weakened sirtuin activity. Several clinical studies are testing NAD^+^ precursors such as nicotinamide riboside (NR) and nicotinamide mononucleotide (NMN). The NADage trial (NCT06208527) is investigating frailty prevention in older adults through endpoints measuring physical performance and molecular markers of NAD^+^ metabolism [164]. Other ongoing studies include NCT02921659, which evaluates NR supplementation in patients with peripheral artery disease using the six-minute walk distance as a functional measure [165]; NCT04691986, which tests NMN supplementation in insulin-resistant individuals with a focus on glucose metabolism and mitochondrial function [166]; and NCT04407390, which investigates NR can attenuate the severity of SARS-CoV-2 infections [167]. Collectively, these trials aim to determine whether NAD^+^ boosters can move beyond biomarker-level effects to achieve clinically meaningful outcomes in populations vulnerable to metabolic decline.

Senolytic therapies, particularly the combination of dasatinib and quercetin (D + Q), have also entered the clinical stage with multiple trials underway. These include NCT04313634, which evaluates epigenetic modifications and systemic markers of aging in older adults; NCT04946383, investigating osteoarthritis with a focus on pain reduction and cartilage biomarkers; NCT04733534, examining functional outcomes and systemic inflammation in frail individuals; and NCT05422885, which explores safety and long-term effects of repeated senolytic dosing [168]. These studies build upon earlier pilot data showing that clearance of senescent cells in patients with idiopathic pulmonary fibrosis improved physical function [11]. However, results in other conditions, such as osteoarthritis, have been inconsistent, underscoring the challenge of translating senolytic efficacy across different tissues and diseases.

Another innovative approach involves supplementation with GlyNAC (glycine plus N-acetylcysteine), aimed at restoring glutathione (GSH) levels, reducing oxidative stress, and improving mitochondrial function in older adults. The clinical trial NCT01870193 has been designed to test these effects and preliminary findings indicate broad multisystem benefits, including improved cognition, muscle strength, walking speed, and reduced inflammation [169]. Notably, Kumar and colleagues provided the most complete evidence through a randomized controlled trial published in Journals of Gerontology: Series A (2022), which demonstrated that GlyNAC supplementation significantly improved mitochondrial function, reduced oxidative stress, lowered systemic inflammation, and enhanced both cognitive and physical performance [10]. These findings support the hypothesis that replenishing glutathione precursors corrects key hallmarks of aging, although the relatively small sample size highlights the need for larger, multicenter validation studies.

Among the completed trials, the MILES study stands out as the first to provide controlled evidence that metformin can induce transcriptional changes consistent with anti-aging signatures in human tissues [155,156]. Specifically, metformin shifted gene expression toward patterns associated with caloric restriction, mitochondrial efficiency, and reduced inflammation. While the study lacked sufficient statistical power to demonstrate clinical endpoints, it remains a milestone in demonstrating biomarker-level modulation by a gerotherapeutic agent.

Taken together, the current body of trials underscores both the progress and limitations of translational geroscience. Rapalogues show promising immune benefits but long-term safety and tolerability remain uncertain, with EVERLAST expected to provide critical data on chronic use in aging populations. Metformin benefits from strong observational and preclinical data, complemented by biomarker evidence from MILES, while the delayed TAME trial remains one of the most highly anticipated large-scale attempts to demonstrate disease-delay via a gerotherapeutic intervention. NAD^+^ boosters, although supported by encouraging mechanistic rationale, have so far shown only modest results, with ongoing studies set to clarify whether these effects can be generalized. Senolytics are conceptually compelling and effective in animal models, but human evidence remains at an early stage, with divergent results suggesting tissue-specific responses and the need for precise patient selection. Finally, GlyNAC is emerging as a promising multi-system intervention with encouraging early-phase results, but its future impact will depend on validation in larger clinical settings.

Overall, these trials illustrate a translational pipeline where candidate molecules progress from mechanistic rationale to early human testing. Most studies remain small, biomarker-focused, or early-phase, reflecting both the novelty of the field and the intrinsic challenges in designing appropriate endpoints for aging interventions (Table 3). Yet the diversity of strategies, spanning rapalogues, metformin, NAD^+^ boosters, senolytics, and redox-restoring compounds like GlyNAC, underscores the maturation of geroscience into a truly translational discipline.

## 8. New Dimensions in Geroscience: Beyond Pharmacology

The evolution of geroscience has been driven largely by pharmacological interventions targeting nutrient-sensing pathways, senescence, or metabolic decline. Yet the field has increasingly recognized that the biology of aging cannot be reduced to druggable targets alone. A truly integrative vision of longevity requires incorporating lifestyle, environmental, regenerative, and digital dimensions, broadening the translational horizon beyond molecules to systems. This chapter explores these new dimensions, highlighting how lifestyle interventions, microbiome modulation, epigenetic reprogramming, regenerative strategies, and digital medicine are reshaping the future of aging science.

In this context, stem cell research represents a central pillar of modern anti-aging and regenerative medicine. Age-related stem cell exhaustion and dysfunction play a direct role in tissue degeneration, impaired repair mechanisms, and functional decline across multiple organ systems. Advances in stem cell–based therapies, organoid technologies, and partial cellular reprogramming are therefore emerging as complementary strategies within the geroscience framework, alongside metabolic, pharmacological, and lifestyle interventions.

Lifestyle factors remain the foundation of healthy aging. Caloric restriction and its variants, such as intermittent fasting and time-restricted feeding, have consistently demonstrated benefits in extending healthspan across multiple model organisms. In humans, observational studies and controlled trials suggest improvements in metabolic flexibility, inflammation, and cardiovascular health [170,171]. Exercise, particularly endurance and resistance training, is equally fundamental, not only maintaining muscle mass and cardiovascular fitness but also exerting molecular effects via AMPK activation, mitochondrial biogenesis, and modulation of systemic inflammation [172]. Sleep quality and circadian alignment are emerging as critical determinants, with disruption linked to accelerated epigenetic aging and increased risk of neurodegeneration [173]. These interventions, while often dismissed as “low-tech,” remain unparalleled in their accessibility and capacity to synergize with pharmacological approaches.

A second frontier is the human microbiome, increasingly recognized as a key regulator of host metabolism, immunity, and even neurocognitive health. Aging is accompanied by loss of microbial diversity, expansion of pro-inflammatory taxa, and diminished production of short-chain fatty acids, all of which contribute to inflammaging [174]. Interventions ranging from dietary fiber and probiotics to fecal microbiota transplantation (FMT) are being tested for their ability to restore a youthful microbial ecology. In preclinical studies, transplantation of young gut microbiota into aged mice reversed immune decline and improved cognitive performance [175]. Early-phase human studies are exploring the feasibility of microbiome-targeted interventions to mitigate frailty and metabolic dysfunction [176]. The microbiome thus represents a malleable and environmentally sensitive component of geroscience, bridging lifestyle and pharmacology.

Epigenetics provides yet another transformative dimension. The discovery of epigenetic clocks, based on DNA methylation signatures, has redefined how biological aging can be measured, enabling sensitive detection of deviations from chronological age [177]. More radically, the field of epigenetic reprogramming suggests that aspects of aging may be reversed at the cellular level. Partial reprogramming using Yamanaka factors has rejuvenated cellular function in mice without inducing dedifferentiation or tumorigenesis [178]. Translational studies are underway to explore whether transient reprogramming can reset epigenetic clocks and restore tissue function in humans [179]. While highly experimental, these approaches could, in principle, complement pharmacological geroprotectors, shifting the focus from delaying aging to actively rejuvenating tissues.

Regenerative medicine intersects naturally with geroscience. Advances in stem cell biology, organoids, and tissue engineering are providing unprecedented tools to restore aged tissues. Hematopoietic stem cell transplantation has already demonstrated rejuvenating effects in experimental settings, while mesenchymal stem cell therapies are being tested in clinical trials for frailty, osteoarthritis, and neurodegenerative conditions [180]. Organoid technology, by recapitulating tissue architecture in vitro, not only provides models for aging research but also paves the way for regenerative implants [181]. Meanwhile, biomaterials and 3D bioprinting offer avenues to replace or repair aged tissues. The synergy between regenerative approaches and geroscience lies in their shared aim: not only extending lifespan but restoring functional capacity across organ systems.

Digital medicine and artificial intelligence add yet another transformative layer. The explosion of longitudinal datasets, including genomics, proteomics, metabolomics, and digital phenotyping, allows for the construction of biological age predictors and the development of “digital twins” of aging individuals [182]. Machine learning algorithms can integrate heterogeneous data streams to identify early markers of decline, personalize interventions, and even simulate the outcomes of geroprotective therapies [183]. For example, AI-based models have been applied to predict biological age trajectories under different lifestyle or pharmacological regimens [184]. Digital geroscience promises to transform longevity research from population-level interventions to highly individualized, data-driven strategies. A conceptual roadmap summarizing these steps is presented in Table 4. These scientific advances are accompanied by profound ethical and societal considerations. Interventions aimed at modifying aging raise questions of accessibility, equity, and justice. The specter of widening health disparities looms if advanced therapies such as reprogramming or stem cell interventions remain restricted to affluent populations [185]. Equally concerning is the risk of over-medicalizing aging, reframing a natural process as a pathology to be “treated.” Critics warn that this perspective could undermine social and psychological adaptations to aging [186]. On the other hand, proponents argue that geroscience should be regarded as a legitimate field of preventive medicine, aiming not at immortality but at compression of morbidity and preservation of dignity in later life [187].

**Figure 5:**
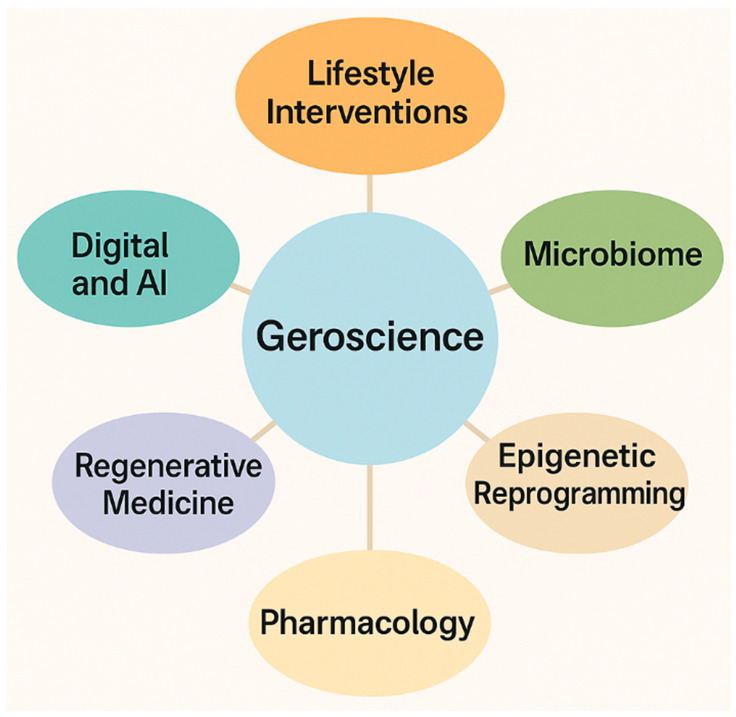
Schematic overview of emerging dimensions in geroscience. Beyond pharmacological approaches, lifestyle, microbiome modulation, epigenetic reprogramming, regenerative medicine, and digital/AI strategies converge as complementary pillars to extend healthspan.

A further challenge lies in defining endpoints. While pharmacological trials may rely on biomarkers or composite morbidity indices, lifestyle and digital interventions require broader measures of success, encompassing quality of life, autonomy, and social engagement [188]. The incorporation of patient-reported outcomes and multidimensional measures of resilience is therefore critical. These issues underscore that geroscience, while biologically driven, must remain anchored in humanistic and societal frameworks. Taken together, these new dimensions lifestyle, microbiome, epigenetics, regenerative medicine, and digital health expand the scope of geroscience beyond pharmacology. They reveal aging as a systemic process, shaped by interactions between biology, environment, and society. Pharmacological interventions such as rapamycin or metformin may remain central, but their effects are amplified when embedded in comprehensive strategies that include diet, exercise, microbiome modulation, and digital monitoring. The convergence of regenerative and AI-driven approaches signals a future where aging interventions are both personalized and multimodal. Ultimately, the challenge for geroscience is integration: combining molecular insights, clinical interventions, lifestyle strategies, and ethical foresight into a cohesive framework. Such integration will not only extend healthspan but also redefine aging itself not as inevitable decline, but as a dynamic and malleable phase of life. The trajectory from mythical elixirs to molecular geroprotectors now evolves further, towards a multidimensional vision where biology, technology, and humanity converge (Figure 5).

## 9. Sex Differences in Aging and Longevity: XX vs. XY at the Crossroads of Biology and Therapy

One of the most intriguing dimensions of human aging lies in the persistent survival gap between women and men. Across nearly all populations worldwide, and consistently in animal models, individuals with two X chromosomes (XX) outlive those with XY karyotypes [189]. This survival advantage, which translates into women living on average four to seven years longer than men, cannot be fully explained by behavioral or sociocultural factors, although these play a role [190]. Instead, it reflects a profound interplay of genetic, hormonal, immunological, and metabolic mechanisms that have become increasingly evident with advances in geroscience.

At the genetic level, the redundancy of the X chromosome appears to provide a biological buffer against aging [191]. While one X chromosome is largely inactivated, a subset of genes escape inactivation, conferring mosaic protection and enhanced resilience against deleterious mutations [192]. In contrast, the Y chromosome is small, gene-poor, and subject to progressive attrition with age. The phenomenon known as mosaic loss of chromosome Y (mLOY), particularly in circulating hematopoietic cells, has emerged as a robust biomarker of aging in men and is strongly associated with increased risk of cardiovascular disease, cancer, neurodegeneration, and overall mortality [193]. The dual X thus appears to serve as a molecular insurance system, whereas loss of Y contributes to the male longevity disadvantage.

Telomere biology also supports sex differences: women generally display longer telomeres and slower erosion rates, correlating with delayed onset of age-related pathologies [194]. Epigenetic aging clocks confirm that men accumulate biological age faster than women, even after controlling for lifestyle and comorbidities [195]. Mitochondrial function follows a similar pattern, with female mitochondria demonstrating superior oxidative phosphorylation, reduced accumulation of reactive oxygen species, and more efficient DNA repair pathways throughout midlife [196]. These cellular advantages may underpin the relative protection observed in women against metabolic and neurodegenerative decline.

Hormonal influences further accentuate this divergence. Estrogens exert protective effects on vascular endothelium, lipid metabolism, bone health, and immune regulation, while also enhancing antioxidant defenses and mitochondrial efficiency [197]. The menopausal transition marks a turning point, coinciding with acceleration in female cardiovascular and metabolic risk [198]. By contrast, testosterone is linked to greater muscle mass and physical performance in younger men, but contributes to pro-inflammatory states, risky behavior, and adverse cardiovascular outcomes later in life [199,200].

The immune system provides another axis of difference. Women exhibit more robust innate and adaptive responses, leading to superior outcomes after infections and vaccinations [201]. However, this enhanced reactivity also underlies their higher prevalence of autoimmune diseases. Men, conversely, experience earlier and more pronounced immunosenescence, with reduced thymic output, impaired T-cell responses, and greater vulnerability to infectious mortality in older age [202]. This “male–female immune paradox” has been highlighted most recently by the COVID-19 pandemic, in which male sex was consistently associated with worse outcomes, despite similar exposure rates [203].

Clinical and epidemiological data confirm these molecular insights. Global analyses show that women’s life expectancy continues to exceed that of men in almost all countries, though the gap is narrowing in high-income settings due to lifestyle convergence [204]. Importantly, women live longer but with a higher burden of multimorbidity and disability in late life, while men often experience earlier mortality but shorter periods of frailty [205]. Recent cohort studies have identified sex-specific trajectories in cardiovascular disease, dementia, and cancer incidence, with estrogen-related pathways implicated in delaying pathology onset in women [206]. Large-scale epigenetic and proteomic studies have further revealed sex-specific signatures of inflammaging, with men exhibiting earlier and more pronounced activation of pro-inflammatory cytokine networks such as IL-6 and TNF-α [207,208].

These biological differences have direct therapeutic implications. The emerging paradigm of precision geroscience recognizes that interventions cannot be “sex-neutral.” Pharmacological strategies such as rapamycin, metformin, senolytics, or NAD^+^ boosters may display distinct efficacy and safety profiles in men and women [209]. For example, caloric restriction and fasting-mimicking diets appear to yield greater metabolic benefits in women in some trials, whereas men may derive stronger improvements in muscle function [210,211]. Hormone-based therapies, particularly estrogen replacement in women, can partially mitigate postmenopausal acceleration of biological aging, though long-term risks remain debated [212,213]. Lifestyle interventions exercise, sleep optimization, and microbiome modulation are universally beneficial, yet evidence suggests sex-specific metabolic and inflammatory responses [172].

**Figure 6:**
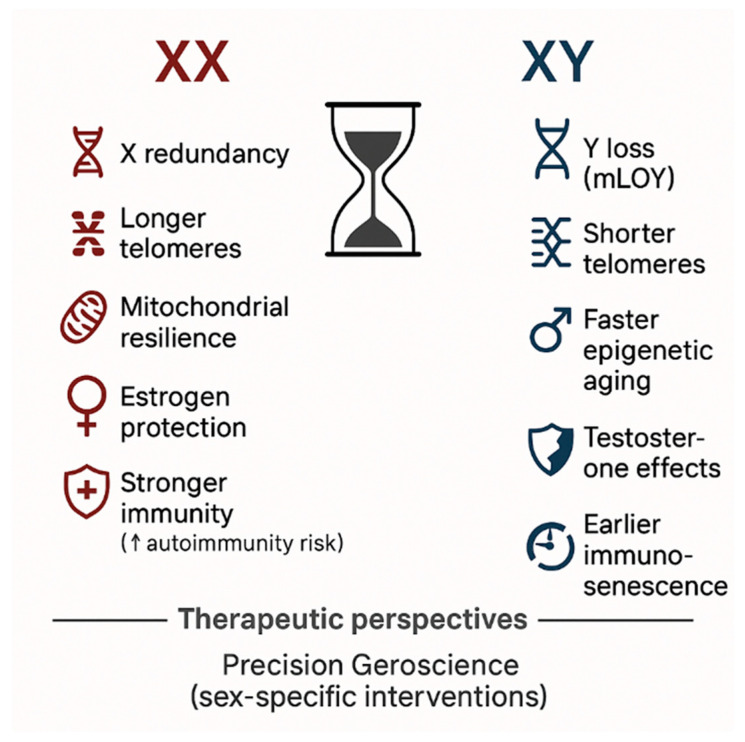
Schematic overview of sex differences in aging and longevity (XX vs. XY), highlighting key genetic, cellular, and hormonal factors, with implications for precision geroscience and sex-specific interventions.

Ultimately, the study of sex differences in aging reveals not only why women tend to live longer, but also why they often do so with a different health profile compared to men. Understanding and harnessing these molecular, hormonal, and immunological differences will be essential for the next generation of longevity medicine (Figure 6). The promise of geroscience lies not merely in extending lifespan, but in tailoring strategies to maximize healthspan in both sexes acknowledging that XX and XY aging are parallel but distinct biological journeys [214,215].

## 10. Conclusions and Future Perspectives in Geroscience

The history of humankind’s search for longevity stretches from mythical elixirs and religious promises of immortality to the modern science of geroscience. Over the past decades, the study of aging has shifted from speculative philosophy to evidence-based biology, unveiling conserved molecular pathways, therapeutic strategies, and translational initiatives that make the prospect of extending human healthspan increasingly tangible. The preceding chapters have examined nutrient-sensing pathways, inflammatory cytokines, clinical trials, and new scientific dimensions beyond pharmacology. Here, we synthesize these insights and explore the ethical, societal, and future implications of geroscience as it transitions from discovery to implementation.

A central conclusion is that aging is no longer perceived as an immutable process. Discoveries involving mTOR, AMPK, and sirtuins have reframed aging as a modifiable biological trajectory. Interventions such as rapamycin, metformin, NAD^+^ precursors, and senolytics have demonstrated the capacity to delay or reverse hallmarks of aging in preclinical models and, increasingly, in human studies. This recognition is profound: it transforms aging into a legitimate target for medical intervention, shifting the paradigm from disease treatment to healthspan preservation [6,31].

At the same time, geroscience is not limited to pharmacology. Lifestyle interventions, caloric restriction, intermittent fasting, exercise, circadian alignment remain the cornerstone of longevity strategies. They interact synergistically with molecular pathways, reinforcing autophagy, mitochondrial biogenesis, and anti-inflammatory responses [171]. The microbiome, epigenetic clocks, regenerative medicine, and digital innovations expand the framework, illustrating that aging is a systemic process influenced by environment, technology, and society [183]. Such multidimensionality ensures that geroscience is not merely a subfield of pharmacology but a comprehensive paradigm integrating biology, medicine, and social science.

The clinical translation of these discoveries is underway but remains in its infancy. Registered trials of rapalogues, metformin, NAD^+^ boosters, senolytics, and GlyNAC illustrate the diversity of approaches under evaluation. Early signals are promising—rapamycin derivatives enhancing vaccine responses, metformin altering aging-related transcriptomes, NAD^+^ boosters improving muscle physiology, senolytics ameliorating fibrosis, and GlyNAC restoring redox balance [9,10,11,130,138]. Yet most studies remain small, early-phase, and biomarker-driven. The challenge lies in moving toward large-scale, long-term trials with clinically meaningful endpoints. The delayed TAME trial is emblematic: its success would provide proof-of-concept that aging itself can be targeted in a regulatory framework [144].

This translation raises profound ethical and societal questions. Should aging be considered a disease? If so, what are the implications for healthcare systems, insurance policies, and societal perceptions of older adults? Critics warn of the dangers of medicalizing aging, transforming a universal life stage into a pathology to be treated [186]. Others argue that failing to address the biological underpinnings of aging would be ethically negligent, as it condemns millions to preventable morbidity. Between these poles lies a nuanced view: geroscience should not be about denying aging, but about compressing morbidity, extending resilience, and preserving dignity in later life.

Equity of access emerges as a pressing concern. Advanced gerotherapeutics such as epigenetic reprogramming, stem cell therapies, or AI-driven personalized interventions may initially be available only to wealthy individuals, exacerbating health disparities. The history of medicine shows that innovations often diffuse unevenly, privileging the affluent before reaching broader populations [34]. If geroscience becomes another vector of inequality, it risks undermining its societal legitimacy. Policymakers and health systems must therefore anticipate issues of cost, accessibility, and fairness. Initiatives to incorporate lifestyle interventions, low-cost drugs like metformin, and public health strategies will be essential to ensure equitable impact.

Another challenge is the definition of clinical endpoints. Unlike disease-specific drugs, geroprotectors aim to delay the onset of multiple conditions simultaneously. Composite outcomes, as pioneered in the TAME trial, offer a solution, but regulators remain hesitant to accept aging itself as a therapeutic indication [216]. Moreover, biomarkers such as epigenetic clocks, proteomic signatures, or immune profiles, while promising, are not yet validated as surrogate endpoints for regulatory approval [16]. Developing robust, standardized measures of biological age will be a prerequisite for mainstream adoption of geroscience interventions.

The societal implications extend beyond healthcare. If healthspan is substantially prolonged, societies will need to rethink retirement policies, labor markets, and intergenerational equity. A world where individuals remain healthy and productive into their eighties or nineties could alleviate the burden of chronic disease and dependency, but it also raises questions about economic sustainability and resource allocation. Conversely, if interventions extend lifespan without proportional gains in healthspan, the societal burden of morbidity would intensify a scenario that must be avoided at all costs.

Public communication and perception represent another critical dimension. Media coverage of anti-aging breakthroughs often oscillates between hype and skepticism, fueling unrealistic expectations or premature adoption of unproven interventions. This dynamic risks eroding trust in legitimate science. Geroscience must therefore cultivate transparency, emphasizing both the potential and the limitations of current evidence. Engaging with patients, caregivers, and policymakers in open dialogue will be essential to align scientific progress with societal values.

Looking forward, the future of geroscience lies in precision and integration. Precision geroscience will tailor interventions to individual biological profiles, informed by genomics, epigenetics, metabolomics, and microbiome signatures [215]. Integration will combine pharmacological, lifestyle, regenerative, and digital strategies into multimodal interventions, much as oncology combines surgery, chemotherapy, targeted therapy, and immunotherapy. Early examples include combinations of rapamycin with MEK inhibitors, or senolytics with NAD^+^ boosters, which demonstrate synergistic effects in animal models [83]. Similar approaches in humans could maximize efficacy while minimizing toxicity.

Emerging frontiers such as epigenetic reprogramming and AI-driven digital twins hint at a future where aging trajectories can not only be slowed but actively reset. Reprogramming studies in mice have already demonstrated reversal of biological age markers and restoration of organ function [217]. Digital twins, constructed from multi-omic and clinical data, could allow simulations of individualized interventions, predicting responses before therapies are applied [182]. These technologies illustrate the trajectory of geroscience toward not only extending healthspan but also reshaping the very concept of aging. The ethical frameworks must evolve in parallel. If longevity interventions become widely available, how will societies ensure they are used to promote equity rather than exacerbate inequality? If digital tools predict aging trajectories, how will privacy, autonomy, and consent be protected? If reprogramming becomes feasible, what limits should be placed on its application? These questions demand multidisciplinary collaboration, bringing together scientists, ethicists, policymakers, and the public.

In conclusion, the trajectory from mythical elixirs to modern geroscience reflects one of the most profound paradigm shifts in medicine. Aging is no longer destiny but a modifiable process, open to scientific inquiry and clinical intervention. The promise of geroscience is not immortality but the compression of morbidity, the extension of healthspan, and the preservation of human dignity in later life. Realizing this promise will require not only rigorous science and clinical innovation but also ethical foresight, equitable policies, and societal engagement. The coming decades will determine whether geroscience fulfills its transformative potential or becomes another unbalanced innovation. What is clear is that the journey has only begun, and the future of aging will be written at the intersection of biology, technology, and humanity.

## Figures and Tables

**Table 1:** Preclinical Models of Aging.

Model Organism	Key Characteristics	Main Uses in Aging Research
Yeast (*Saccharomyces cerevisiae*)	Very short replicative lifespan; easy genetic manipulation; conserved TOR & sirtuin pathways	Discovery of nutrient-sensing pathways (TOR, sirtuins); high-throughput drug screening
Nematode (*C. elegans*)	~20 days lifespan; ~60% gene homology with humans; transparent body	Identification of >400 longevity genes; stress resistance; senotherapeutic screening
Drosophila (*D. melanogaster*)	Lifespan ~60–80 days; powerful genetics; well-mapped organs	Indy gene longevity models; neural senescence studies; metabolic regulation
Fish (Killifish, Zebrafish, Axolotl)	Killifish lifespan 3–6 months; transparent zebrafish embryos; axolotl regeneration	Rapid drug testing; vertebrate regeneration models; transient senescence in repair
Rodents (mice, rats)	Lifespan 2–3 years; advanced genetic tools; disease models	Caloric restriction; rapamycin studies; GH/IGF-1 modulation; senescence clearance; frailty models
Swine (mini-pigs)	Strong physiological similarity to humans; cardiovascular & metabolic resemblance	Atherosclerosis & metabolic aging models; cardiovascular aging; translational testing of interventions
Non-human primates (macaques, marmosets)	High similarity to human physiology; age-related pathologies	Long-term CR studies; immune/metabolic aging; preclinical translational research
Human stem-cell–derived organoids	3D human-specific tissues modeling aging features (stem-cell exhaustion, mitochondrial dysfunction, DNA damage)	High capture human aging signatures better than animal models

**Table 3:** Clinical Trials in Geroscience.

Molecule/Intervention	ClinicalTrials.Gov ID (NCT)	Description/Status	Main Findings/Notes
Rapalogues (Everolimus–mTORC1)	NCT05835999	EVERLAST–safety and anti-aging effects (24 weeks, ongoing)	Awaiting results; focus on immunosenescence and safety.
Metformin	NCT02432287	MILES—biomarkers of aging (completed, preliminary results)	Transcriptomic shifts consistent with anti-aging; no definitive clinical outcomes yet.
NAD^+^ boosters (NR, NMN)	NCT06208527, NCT02921659, NCT04691986, NCT04407390	NADage and others—frailty, NAD^+^ metabolism, muscle function (ongoing)	Early data suggest improved vascular/muscle function; modest overall effects.
Senolytics (Dasatinib + Quercetin)	NCT04313634, NCT04946383, NCT04733534, NCT05422885	Cellular senescence, epigenetics, physical function (ongoing)	Pilot study in IPF showed improved function; mixed outcomes in OA.
GlyNAC (Glycine + NAC)	NCT01870193	Pilot RCT—glutathione, oxidative stress, mitochondrial function (completed, promising results)	J Gerontol A 2022: improved GSH, mitochondrial function, inflammation, cognition, strength.

**Table 4:** Roadmap for integrating combination gerotherapeutics with digital health.

Step	Description
1. Digital biomarker profiling & wearable phenotyping	Acquisition of continuous physiological, behavioral, and functional data through wearables, sensors, and smartphone-based monitoring.
2. Multi-omic & functional stratification	Integration of genomics, epigenomics, metabolomics, proteomics, and functional tests to classify individuals into biologically meaningful risk/aging clusters.
3. Personalized combination therapy	Selection and implementation of multi-target interventions (senolytics, metabolic modulators, NAD^+^ boosters, lifestyle therapies) tailored to the individual profile.
4. AI-assisted monitoring & adaptive adjustment	Real-time tracking of responses with algorithm-driven adjustment of dosages, combinations, or timing based on predictive analytics.
5. Long-term evaluation & clinical outcomes	Assessment of safety, adherence, functional improvements, and reduction in biological age trajectories using integrated digital platforms.

## Data Availability

No new data were created or analyzed in this study. Data sharing is not applicable to this article.

## Glossary

**Term**	**Definition**
AMPK (AMP-activated protein kinase)	Cellular energy sensor promoting catabolic pathways, enhancing autophagy and stress resistance.
Autophagy	Catabolic process degrading damaged proteins and organelles.
Caloric Restriction (CR)	Reduced caloric intake without malnutrition; extends lifespan.
Epigenetic Clocks	DNA methylation-based predictors of biological age.
Geroscience	Field linking aging biology with chronic diseases.
GlyNAC	Glycine + NAC combination restoring glutathione and reducing oxidative stress.
mTOR	Regulator of growth, protein synthesis, and nutrient sensing.
NAD+	Coenzyme essential for mitochondrial function; declines with age.
NAD+ Boosters	Compounds increasing NAD+ levels (NR, NMN).
Rapalogues	Rapamycin analogs with distinct PK/safety profiles.
SASP	Inflammatory secretome of senescent cells.
Senescent Cells	Cells in permanent cycle arrest producing SASP.
Senolytics	Drugs eliminating senescent cells selectively.
Sirtuins	NAD+-dependent enzymes regulating metabolism and aging.
Telomeres	Repetitive DNA ends shortening with cell division.
Telomerase	Enzyme maintaining telomere length.
Fasting-Mimicking Diets	Dietary protocols mimicking fasting effects.
Nutrient-Sensing Pathways	mTOR, AMPK, sirtuins, IGF-1 regulating lifespan.
IGF-1/GH axis	Hormonal axis involved in growth and longevity.
FOXO transcription factors	Regulators of stress resistance and longevity.
DNA methylation drift	Stochastic age-related methylation changes.
Inflammaging	Chronic low-grade inflammation increasing with age.
Mitophagy	Autophagic removal of damaged mitochondria.
Proteostasis	Maintenance of protein homeostasis.
Senomorphic drugs	Agents suppressing SASP without killing cells.
Autophagy flux	Efficiency of autophagic degradation.
Epigenetic reprogramming	Partial resetting of epigenetic marks.
Geroprotectors	Interventions slowing biological aging.
Polyphenols/antioxidants	Plant compounds modulating oxidative stress.
Xenobiotics	Foreign substances requiring detoxification.
ADME	Absorption, Distribution, Metabolism, Excretion.
Half-life	Time for plasma concentration to halve.
Bioavailability	Fraction of active substance reaching circulation.
Pathway intermediates	Molecules mediating signaling events.
Biomarkers (proteomic/metabolomic)	Molecular signatures of aging.
Sex-specific differences	Aging and treatment differences by sex.
Metformin mechanisms	Actions via AMPK activation and mitochondrial effects.
mTORC1 vs. mTORC2	Distinct complexes regulating growth and insulin signaling.
TOR inhibitors (2nd generation)	Selective mTOR inhibitors with improved profiles.
